# Editorial for the Special Issue “The Role of Dietary Bioactive Compounds in Human Health” in *Molecules*

**DOI:** 10.3390/molecules30102128

**Published:** 2025-05-12

**Authors:** Bin Du, Baojun Xu

**Affiliations:** 1 Hebei Key Laboratory of Natural Products Activity Components and Function, Hebei Normal University of Science and Technology, Qinhuangdao 066004, China; bindufood@aliyun.com; 2 Food Science and Technology Program, Department of Life Sciences, Beijing Normal-Hong Kong Baptist University, Zhuhai 519087, China

The prevalence of chronic diseases, such as cardiovascular disease, diabetes, chronic obstructive pulmonary disease and severe mental health disorders, has been constantly increasing over the last two decades [1]. It has been recognized that greater adherence to the dietary advice of specialists is a critical component in preventing and managing chronic diseases. Bioactive compounds are ingredients obtained from natural raw materials (e.g., plants, fruits, vegetables, grains, meat, or seafood). Dietary bioactive compounds play an important role in the daily lives of consumers as they are shown to improve health [2]. However, the molecular mechanisms behind the anti-tumor, anti-inflammatory and antioxidant activities of these compounds are not quite clear yet.

This Special Edition is aimed at collecting and summarizing the existing knowledge on the disease prevention effects (including antioxidative, anti-tumor, anti-obesity, anti-diabetes, anti-inflammatory, cardiovascular-protective, skin-protective, and neuroprotective activities, etc.) of dietary bioactive compounds. This Special Issue includes eleven papers covering the above-mentioned aspects, including six reviews and five research papers.

Saponins are a diverse group of compounds that are widely distributed throughout the plant kingdom, and are characterized by their structures containing a triterpene or steroid aglycone and one or more sugar chains [3]. Saponins have various pharmacological activities. Alzheimer’s disease (AD), a neurodegenerative disorder, presents a significant global health burden. Saponins have displayed therapeutic potential in treating Alzheimer’s disease. Moussa et al. investigated the binding potential of saponins from *Platycodon grandiflorum* with six AD-related proteins using computational and quantum chemistry simulations (Contribution 1). Molecular docking revealed interactions between saponins and key AD enzymes, including GSK-3β and synapsins I–III. MD simulations highlighted three compounds—polygalacin D2, polygalacin D, and platycodin D—showing binding profiles comparable to standard drugs (ifenprodil and donepezil). Further DFT analysis elucidated their electronic properties and anti-AD potential.

Phenolic compounds are a large group of plant metabolites that have long attracted interest from researchers worldwide due to their functions in human health and well-being [4]. In Contribution 2 of this Special Issue, polyphenol and caffeine levels in popular types of coffee from six Warsaw-based franchises (120 samples total) were analyzed. A strong positive correlation was found between polyphenol and caffeine contents (Contribution 2).

In another contribution to this Special Issue, the authors explored whether esculetin can alleviate H_2_O_2_-induced apoptosis and pyroptosis in HepG2 cells. The results showed that esculetin pretreatment significantly improved cell viability, reduced ROS levels, and suppressed apoptosis by modulating the expression of Bax, Bcl-2, cleaved Caspase-3, cleaved PARP, and Cyt-c. It also attenuated pyroptosis by lowering LDH release and the levels of NLRP3, cleaved Caspase-1, IL-1β, and GSDMD-N. Mechanistically, esculetin inhibited the JNK pathway (p-JNK, p-c-Fos, p-c-Jun), and its protective effects were reversed by the JNK activator anisomycin (Contribution 3).

Furthermore, Wu et al. developed an extraction method combining hot water extraction with three-phase partitioning, followed by ultrasonic degradation to produce ultrasonic *Laminaria japonica* polysaccharides (ULJPs). A comparative analysis revealed that ULJPs had a lower molecular weight (153 kDa) and a more porous structure than LJPs, while maintaining similar primary structures (α-hexopyranoses with 1,4-glycosidic bonds). Notably, ULJP exhibited enhanced bioactivity (Contribution 4).

While sodium butyrate (NaB) is recognized for its therapeutic potential, its effects on macrophage-mediated inflammation remain unclear. One study investigated NaB’s protective mechanisms against AGEs-induced damage in THP-1 macrophages through PI3K-dependent autophagy pathways (Contribution 5).

There is one review related to functional foods in this Special Issue. Flaxseeds, valued for millennia, are gaining modern recognition for their rich nutritional profile, including omega-3 fatty acids, lignans, protein, and fiber. These components contribute to diverse health benefits, such as cardiovascular protection, cancer prevention, blood sugar regulation, digestive health, and weight management. As a functional food, flaxseeds offer significant health advantages but continued research and technological advancements are required to optimize their benefits while minimizing risks (Contribution 6). Another contribution examines the unique characteristics and applications of Bactrian camel (BC) milk, an emerging functional food with distinct advantages over other mammalian milk. As interest grows in BC milk’s functional food applications, this synthesis provides essential guidance for research and commercial development in this promising sector. The contribution highlights both the scientific basis and economic opportunities surrounding BC milk utilization (Contribution 7).

UV-induced skin photoaging disrupts skin metabolism and impacts well-being. While conventional treatments often cause side effects, natural compounds like epigallocatechin gallate (EGCG), the primary bioactive in tea polyphenols, offer safer alternatives. Another contribution to this Special Issue examines EGCG’s mechanisms and benefits and innovations regarding its delivery for photoaging repair (Contribution 8).

Phytochemicals in plant-based foods, particularly polyphenols, demonstrate significant medicinal value despite being non-essential nutrients. Contribution 9 examines their cardioprotective effects through three key mechanisms: oxidative stress regulation, vascular protection, and anti-inflammatory action (Contribution 9).

*Sambucus nigra* L. (elderberry) has been traditionally valued for its medicinal properties, attributed to its rich antioxidant content. Contribution 10 evaluates the anti-inflammatory and anticancer effects of extracts and components of *S. nigra* L. flowers and fruits (Contribution 10).

There is one systematic review of fruits and vegetables included in this Special Issue. Fruits and vegetables not only serve as nutritional sources but also as natural therapeutics, containing bioactive compounds like hirsutine (HSN), an indole alkaloid found in Uncaria species and various foods (e.g., seafood, grains, and fruits such as bananas and citrus). This contribution synthesizes the current evidence (up to July 2023) from PubMed, Scopus, and other databases on HSN’s pharmacological mechanisms and biopharmaceutical properties (Contribution 11).

In summary, the results of the above-mentioned studies will fill the gap between our knowledge of natural bioactive compounds and their underlying cellular signaling and molecular mechanisms. Further studies are needed to discover the cross-connections between oxidative stress and disease prevention in signaling pathways networks.
